# Hepatoprotective effect of protocatechuic acid against type 2 diabetes-induced liver injury

**DOI:** 10.1080/13880209.2023.2181359

**Published:** 2023-05-02

**Authors:** Kaixia Xu, Guang Lu, Qianjin Feng, Shuangchao Chen, Yonghui Wang

**Affiliations:** Basic Medical School, Shanxi University of Chinese Medicine, Shanxi Province, China

**Keywords:** NF-κB pathway, Wnt1/ β-catenin pathway, oxidative stress, inflammation, lipid accumulation

## Abstract

**Context:**

Protocatechuic acid (PCA) has a protective effect on alcoholic liver injury, but the role of PCA in type 2 diabetes-induced liver injury is not well known.

**Objectives:**

This study explores the therapeutic effect and potential mechanism of PCA on type 2 diabetes-induced liver injury.

**Materials and methods:**

An insulin resistance/type 2 diabetic (IR/D) model was established by high-fat diet for 4 weeks + streptozotocin (35 mg/kg; i.p) in male Wistar rats pretreated with or without PCA (15 or 30 mg/kg for 6 d).

**Results:**

PCA at 15 and 30 mg/kg significantly upregulated the levels of body weight (BW; 230.2, 257.8 g), high density lipids (22.68, 34.78 mg/dL), glutathione (10.24, 16.21 nmol/mg), superoxide dismutase (21.62, 29.34 U/mg), glucagon-like peptide-1, glucose transporter-4, Wnt1, and β-catenin, while downregulating those of liver weight (LW; 9.4, 6.7 g), BW/LW (4.1, 2.6%), serum glucose (165, 120 mg/dL), serum insulin (13.46, 8.67 μIU/mL), homeostatic model assessment of insulin resistance (5.48, 2.57), total cholesterol (68.52, 54.31 mg/dL), triglycerides (72.15, 59.64 mg/dL), low density lipids (42.18, 30.71), aspartate aminotransferase (54.34 and 38.68 U/L), alanine aminotransferase (42.87, 29.98 U/L), alkaline phosphatase (210.16, 126.47 U/L), malondialdehyde (16.52, 10.35), pro-inflammatory markers (tumor necrosis factor α (TNF-α (149.67, 120.33 pg/mg)) , IL-6 (89.79, 73.69 pg/mg) and IL-1β (49.67, 38.73 pg/mg)), nuclear factor kappa B (NF-κB), and interleukin-1β, and ameliorated the abnormal pathological changes in IR/D rats.

**Discussion and conclusion:**

PCA mitigates the IR, lipid accumulation, oxidative stress, and inflammation in liver tissues of IR/D rats by modulating the NF-κB and Wnt1/β-catenin pathways.

## Introduction

Diabetes mellitus is a severe chronic metabolic disease arising from defective insulin secretion and/or insulin resistance (IR), with the domain manifestation of the persistently elevated blood glucose level (Ma 2018; Banday et al. 2020). Clinically, diabetes is mainly divided into type 1 diabetes, type 2 diabetes and gestational diabetes (Khan et al. 2019). Notably, type 2 diabetes is the most common type of diabetes worldwide, which is characterized by hyperglycemia, IR and impaired islet cell function (Pearson 2019). As an endocrine organ, the liver plays a critical role in metabolic homeostasis, such as glucose and lipid metabolism, because it is the main site for the metabolism of macronutrients in daily (Maude et al. 2021). With regard to hepatic IR, it is featured as insulin-stimulated suppression in signal transduction pathways for glucose production in the liver (Petersen and Shulman 2018). Chronic hyperglycemia in type 2 diabetes is closely related to the accumulation of triglycerides (TG), the activation of lipogenic enzymes and the induction of endoplasmic stress in hepatocytes, which eventually leads to steatosis and cell death (Loria et al. 2013; Mota et al. 2016). Furthermore, oxidative stress may play an important role in the pathogenesis and progression of diabetic complications. Hyperglycemia promotes the development of IR and impaired insulin secretion through inducing excessive production of reactive oxygen species (ROS) and oxidative stress (Papachristoforou et al. 2020). To this end, reducing the complication of liver injury in type 2 diabetes is as important as reducing the blood glucose level and oxidative stress in long-term treatment.

Protocatechuic acid (PCA) is a phenolic acid widely present in medicinal materials, vegetables and fruits (Wang et al. 2022). Modern pharmacological researches show that PCA possesses multiple biological activities such as antibacterial (Chao and Yin 2009), antiinflammation (Lende et al. 2011), anti-virus (Zhou et al. 2007), antioxidation (Shi et al. 2006), hepatoprotection (Fu et al. 2019), cardioprotection (Bai et al. 2021), etc. Concretely, PCA can suppress osteoclast differentiation and induce the apoptosis of mature osteoclasts (Wu et al. 2016), promote the angiogenesis of human brain microvascular endothelial cells (Kang et al. 2013), and ameliorate lipopolysaccharide-induced septic lung injury by inhibiting oxidative stress, inflammation and apoptosis (Alsharif et al. 2021). Apart from these, PCA also has the ability to improve the cardiac hypertrophy induced by isoproterenol (Bai et al. 2021), and mitigate high glucose-induced extracellular matrix accumulation in diabetic nephropathy (Ma et al. 2018). Furthermore, the hepato-protection function of PCA has been gradually reported. In great detail, PCA restrains the oxidative insults, apoptosis, and inflammation in the liver and kidney tissues of rats with monosodium glutamate intoxication (Kassab et al. 2022). What’s more, PCA can protect the liver against chemically induced fibrosis both *in vitro* and *in vivo* (Cui et al. 2021) and against alcoholic liver injury *via* inhibiting the oxidant pathway (Fu et al. 2019). However, whether PCA also makes a hepatoprotective impact upon type 2 diabetes-induced liver injury through regulating oxidative and inflammation insults requires further exploration.

Once the NF-κB signaling pathway is activated, the IκB kinase IKK will be activated for phosphorylating and ubiquitinating the NF-κB inhibitory protein IκBα, thereby decreasing the cytoplasmic IκBα content. Hence, the NF-κB p65 subunit enters the nucleus from suppressed to activated condition, and promotes the expression of multiple inflammatory factors, resulting in liver disorder (Zheng et al. 2020). In IR/type 2 diabetic (IR/D) rats, the activation of NF-κB is proved to promote the transcription of inflammatory cytokines including IL-1β, which is involved in the development of IR/D by suppressing the translocation of GLUT4 (Abo El-Nasr et al. 2020; Elias-Oliveira et al. 2020; Esmaeilzadeh et al. 2020). Also, the current review states NF-κB as a promising therapeutic target for the probable management of type 2 diabetes (Meyerovich et al. 2018; Bhardwaj et al. 2020). In mammals, the Wnt/β-catenin signaling pathway is mainly composed of Wnt signaling transduction in the membrane, regulation of β-catenin stabilization in the cytoplasm and activation of Wnt target genes in the nucleus (Huang et al. 2019). The Wnt1/β-catenin pathway plays a critical role in liver diseases and can regulate the oxidative stress in hepatic fibrosis (Hasan et al. 2017). Furthermore, NF-κB upregulation and Wnt1/β-catenin pathway inhibition in IR/D rats are blocked by gallic acid (Bashar et al. 2021).

Herein, an IR/D model was established in rats pretreated with or without PCA, so as to investigate the potential role of PCA in IR/D-induced liver injury and decipher the underlying mechanism.

## Materials and methods

### Ethics statement

Experiments using rats were approved by the China Council on Animal Care and Use and authorized by the Shanxi University of Chinese Medicine Medical Ethics Committee (2020DW215). All procedures in this project were performed in the Shanxi University of Chinese Medicine.

### IR/D rat model establishment

Total 24 male Wistar rats after weaning were bought from BIOMARS (Beijing, China). All rats were kept under specific pathogen-free condition, with free access to food and water. Before experiments, all rats were subjected to adaptive feeding for 5 d. Thereafter, the IR/D model was established according to the previous research with minor modification (Abo El-Nasr et al. 2020). Then, the rats were divided into four groups (*n* = 6): the control, the vector, the 15, and the 30 mg/kg PCA groups. Rats in the control group were fed with normal diet for the whole process of the animal experiments. Rats in the 15 mg/kg, 30 mg/kg, and vector groups were firstly injected intraperitoneally with 15 mg/kg PCA (99.99% purity, HY-N0294, MedChemExpress, Princeton, NJ, USA), 30 mg/kg PCA, and an equal volume of normal saline (IN9000, Solarbio, Beijing, China), respectively, lasting for six days. Then, rats in the three groups were additionally subjected to four consecutive weeks of feeding with a high-fat diet (HFD; ASHF3, BIOMARS) *ad libitum* for the establishment of the IR/D model. After that, the rats in the above three groups were fasted overnight and were intraperitoneally injected with 2% 35 mg/kg streptozotocin (STZ; HY-13753, MedChemExpress) which was prepared using 0.1 mmol/L sodium citrate buffer (C1013, Solarbio) once. IR/D was confirmed by measuring fasting levels of glucose and insulin a week after the injection of STZ. The rats with blood glucose level between 200 and 350 mg/dL, hyperlipidemia, and hyperinsulinemia were considered as IR/D and were used in the study.

Then, the rats in the above three groups were fed with a normal diet for a week. On the last day, after the rats were anesthetized with isoflurane (R510-22, RWD, Shenzhen, China), the blood, liver tissue, and terminal ileum of all rats were collected for later use. Meanwhile, the body weight (BW) and liver weight (LW) of all rats were documented.

### Determination of HOMA-IR index

After collection of the serum from all rats, the serum glucose and insulin were firstly detected adopting a blood glucose detection kit (M055162, MREDA, Beijing, China) and a rat insulin Enzyme-linked Immunosorbent Assay (ELISA) kit (M054117, MREDA, Beijing, China) according to the manufacturer’s instructions. Then, the HOMA-IR index was calculated using the formula: HOMA-IR index = insulin level (µIU/mL) × glucose level (mg/dL)/405 (Abo El-Nasr et al. 2020).

### Colorimetry assay

Briefly, the levels of total cholesterol (TC), TG, aspartate aminotransferase (AST), alanine aminotransferase (ALT) and alkaline phosphatase (ALP) in the rat serum were detected by colorimetry assay using TC detection kit (G0113, XiTang Biotechnology, Shanghai, China), TG detection kit (G0450, XiTang Biotechnology), AST detection kit (G0072, XiTang Biotechnology), ALT detection kit (Kang Lang Biological, Shanghai, China, http://www.shklsw.com/shklsw-Products-22288886/), ALP detection kit (Kang Lang Biological, http://www.shklsw.com/shklsw-Products-21955133/).

### ELISA

The levels of low-density lipids (LDL), high-density lipids (HDL), and pro-inflammatory markers (tumor necrosis factor α (TNF-α), IL-6 and IL-1β) in the rat serum, as well as glutathione (GSH) content, superoxide dismutase (SOD) activity and malondialdehyde (MDA) level in rat liver tissues, were detected by ELISA using LDL ELISA kit (M053549, MREDA), HDL ELISA kit (M053550, MREDA), TNF-α ELISA kit (SEKR-0009, Solarbio), IL-6 ELISA kit (SEKR-0005, Solarbio), IL-1β ELISA kit (SEKR-0002, Solarbio), GSH ELISA kit (F15488, XiTang Biotechnology), SOD ELISA kit (F16742, XiTang Biotechnology), and MDA ELISA kit (F16194, XiTang Biotechnology).

### Hematoxylin-eosin staining

Pathological changes in rat liver tissues were examined through a hematoxylin-eosin staining kit (C0105M, Beyotime, Shanghai, China). Simply put, rat liver tissues were firstly incubated with 4% tissue fixative (P1110, Solarbio), xylene (B50009, MERYER), and gradient alcohol. Then, the tissues were embedded into paraffin (YA0012, Solarbio) and cut into 4 μm tissue slices, followed by dewaxing. After being colored with hematoxylin for 10 min, the tissue slices were incubated with hydrochloric acid alcohol (C0163M, Beyotime) for 2 s. Next, the tissue slices were stained with eosin for 1 min, followed by incubated with xylene and neutral gum (IH0265, Leagene, Beijing, China). Finally, the pathological changes of the tissues were observed under a THUNDER tissue microscope (Leica, Weztlar, Germany) with a magnification × 400. Steatosis areas were quantified by with a computerized image analysis system (ImageJ). The steatosis score was assigned under double-blinded conditions to determine the degree of steatosis. The scores were: 0 (<5%), 1 (≥5-33%), 2 (≥33-66%), and 3 (≥66%) (Kleiner et al. 2005). At least 10 fields of view of per sample were evaluated in each group.

### Immunohistochemistry assay

The expression of glucagon-like peptide-1 (GLP-1) in the terminal ileum of rats was analyzed through an immunochemistry assay. Briefly, the tissues of the terminal ileum were first incubated with 4% tissue fixative, xylene, and gradient alcohol. Then, the tissues were embedded into the paraffin and cut into 4 μm tissue slices, followed by dewaxing and incubation with antigen retrieval buffer (C1032, Solarbio) and goat serum (16210064, Thermo, Waltham, Massachusetts, USA). Subsequently, the tissue slices were cultivated with anti-GLP-1 antibody (ab111125, Abcam, Cambridge, UK) at 4 °C overnight and further incubated with rabbit IgG (ab172730, Abcam) at room temperature for 1 h. Next, the tissue slices were stained with DBA buffer (SFQ004, 4 A Biotech, Beijing, China). After that, the tissue slices were further colored with hematoxylin and incubated with gradient alcohol, xylene, and neutral gum. Finally, the GLP-1 positive cells in the tissue slices were observed using the THUNDER tissue microscope with magnification × 100.

### Western blot

Total proteins in the rat liver tissues were firstly extracted using a protein extraction kit (BC3710, Solarbio), followed by concentration quantification using a BCA protein assay kit (PC0020, Solarbio). Then, the proteins were incubated with loading buffer (P1040, Solarbio) at 100 °C for 5 min. After being separated by SDS-PAGE gel (P1200, Solarbio), the proteins were transferred to a PVDF membrane (KGP114, KeyGEN, Nanjing, China). Then, the membrane was blocked with 5% milk without fat for 2 h and incubated with primary antibodies overnight at 4 °C and secondary antibody at room temperature for 2 h. Finally, the protein bands in the membrane were detected under the ChemiDoc MP Chemiluminescence Detector (Bio-Rad, Hercules, CA, USA) with the help of West Femto ECL detection reagent (PE0030, Solarbio). All antibody information was as follows: glucose transporter-4 (GLUT4; 1:500, #2213, Cell Signaling Technology (CST), Boston, MA, USA), nuclear factor kappa B (NF-κB; #8242, CST), Wnt-1 (27935-1-AP, Proteintech, Wuhan, China), β-catenin (1:3000, #8480, CST), interleukin (IL)-1β (26048-1-AP, Proteintech), β-actin (#4970, CST), goat-anti-rabbit IgG (#7074, CST) and goat-anti mouse IgG (#7076, CST).

### Quantitative real-time PCR (qRT-PCR) assays

Total RNA in the rat liver tissue was firstly extracted by an RNA extraction kit (KGR203, KeyGEN), followed by concentration quantification using a UV spectrophotometer (NanoDrop, Thermo). Then, the RNA was used to synthesize cDNA through a reverse transcription kit (KGA1401, KeyGEN). Next, the cDNA was added into Real-time PCR Master Mix (KGA1339-1, KeyGEN) containing relative gene primers (Table 1). Finally, the amplification reaction was performed under QuantStudio 6System (Applied Biosystems, Waltham, Massachusetts, USA). The amplification reaction condition was set in line with the manufacturer’s instructions. Primer sequences for qRT-PCR were obtained from the reported literature (Cai et al. 2020; Bashar et al. 2021).

**Table 1.** Primers of quantitative real-time PCR.

Target gene	Forward primers, 5′–3′	Reverse primers, 5′–3′
GLUT4	ACAATGTCTTGGCTGTGCTG	TCCCACATACATAGGCACCA
NF-κB	AAAAACGCATCCCAAGGTGC	AAGCTCAAGCCACCATACCC
Wnt1	CCCCGTGACCTCTCTGTGTATCAC	TGAAGCCCAGGTGTGGTGGTT
β-catenin	AAGT TCT TGGCTAT TACGACA	ACAGCACCTTCAGCACTCT
IL-1β	GGGATGATGACGACCTGCTAG	ACCACTTGTTGGCTTATGTTCTG
β-actin	CCTCATGCCATCCTGCGTCTG	TTGCTCGAAGTCTAGGGCAACAT

### Statistical analysis

Data in this research were presented as Mean ± Standard Deviation (SD). *p* < 0.05 was deemed as statistical significance. All data were analyzed using one-way analysis of variance (ANOVA) with Tukey’s *post hoc* test in Graphpad 8.0 software.

## Results

### PCA ameliorated IR/D-induced increases in levels of BW/LW, serum glucose, serum insulin, and HOMA-IR in rats

**Figure 1.**
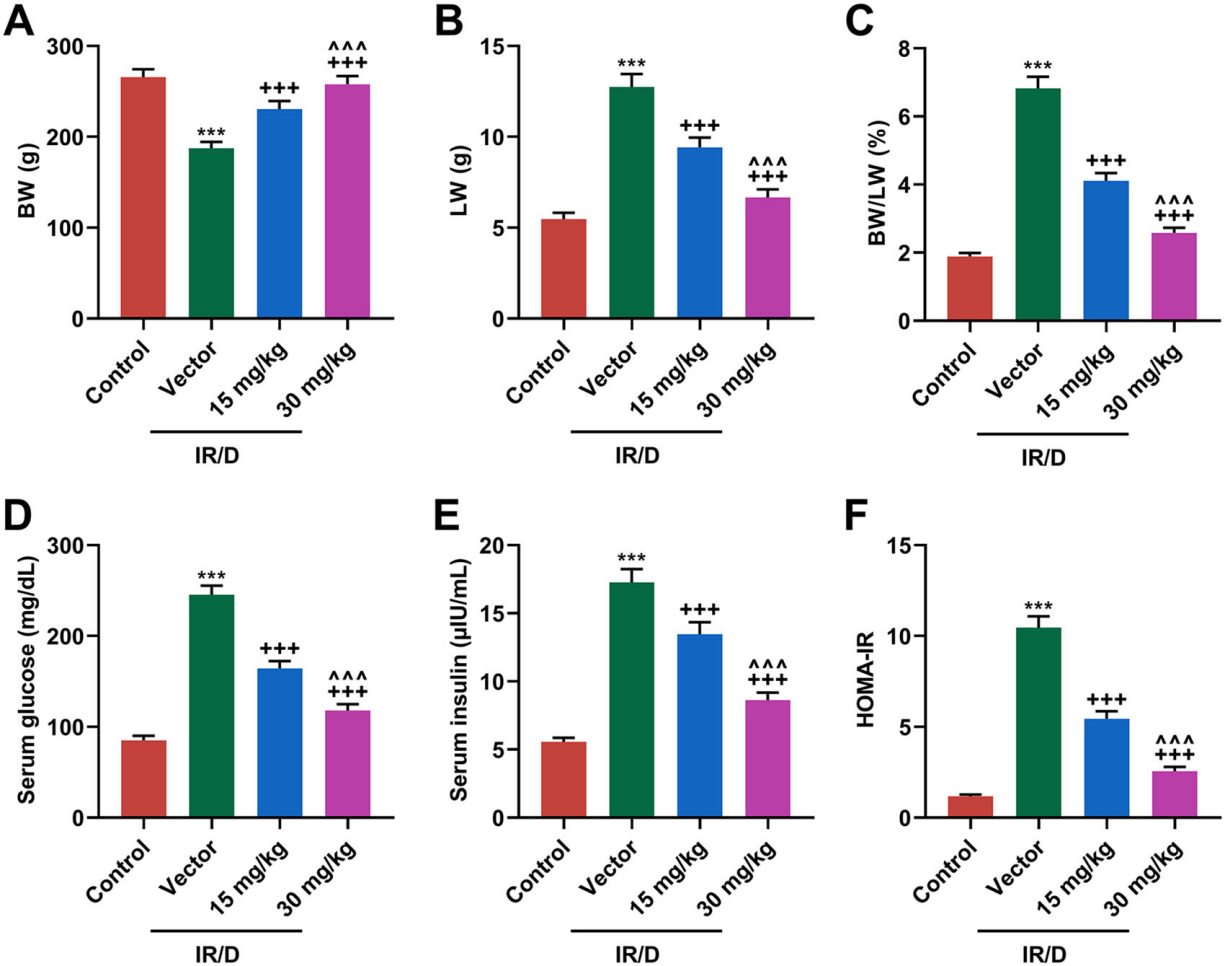
PCA ameliorated IR/D-induced increases in levels of BW/LW, serum glucose, serum insulin, and HOMA-IR in rats. (A–C) The levels of BW (A), LW (B), and BW/LW (C) in IR/D rats pretreated with PCA or not. (D and E) The levels of serum glucose (D) and insulin (E) in IR/D rats pretreated with PCA or not were examined by colorimetry and ELISA. (F) The value of HOMA-IR in IR/D rats pretreated with PCA or not. (***p < 0.001 vs. Control; ^+++^*p* < 0.001 vs. Vector; ^∧∧∧^*p* < 0.001 vs. 15 mg/kg). (PCA: Protocatechuic Acid, IR/D: insulin resistance type 2 diabetic, BW: body weight, LW: liver weight).

After the establishment of the IR/D rat model and then pretreatment with PCA, the BW and LW of rats were documented. As shown in Figure 1(A and B), the level of BW was decreased, but that of LW was increased in IR/D rats as compared to those in the control rats (*p* < 0.001). However, PCA pretreatment upregulated the decreased BW yet downregulated the increased LW of IR/D rats (*p* < 0.001). Meanwhile, the BW/LW ratio was elevated in IR/D rats when compared with that in the control rats (*p* < 0.001, Figure 1(C)), which however was attenuated by PCA pretreatment (*p* < 0.001, Figure 1(C)). The IR of experimental rats was also examined. As illustrated in Figure 1(D and E), the serum glucose (Figure 1(D)), serum insulin (Figure 1(E)), and HOMA-IR (Figure 1(F)) of IR/D rats were all dwindled in comparison with those of the control group rats (*p* < 0.001), while PCA pretreatment ran inversely (*p* < 0.001). Furthermore, compared to 15 mg/kg PCA treatment on IR/D rats, 30 mg/kg PCA treatment exerts a stronger effect on the above indicators (Figure 1, *p* < 0.001). All these results indicated that PCA inhibited the levels of BW/LW and IR of IR/D rats.

### PCA mitigated the abnormal serum levels of liver enzymes and lipid profile in IR/D rats

**Figure 2.**
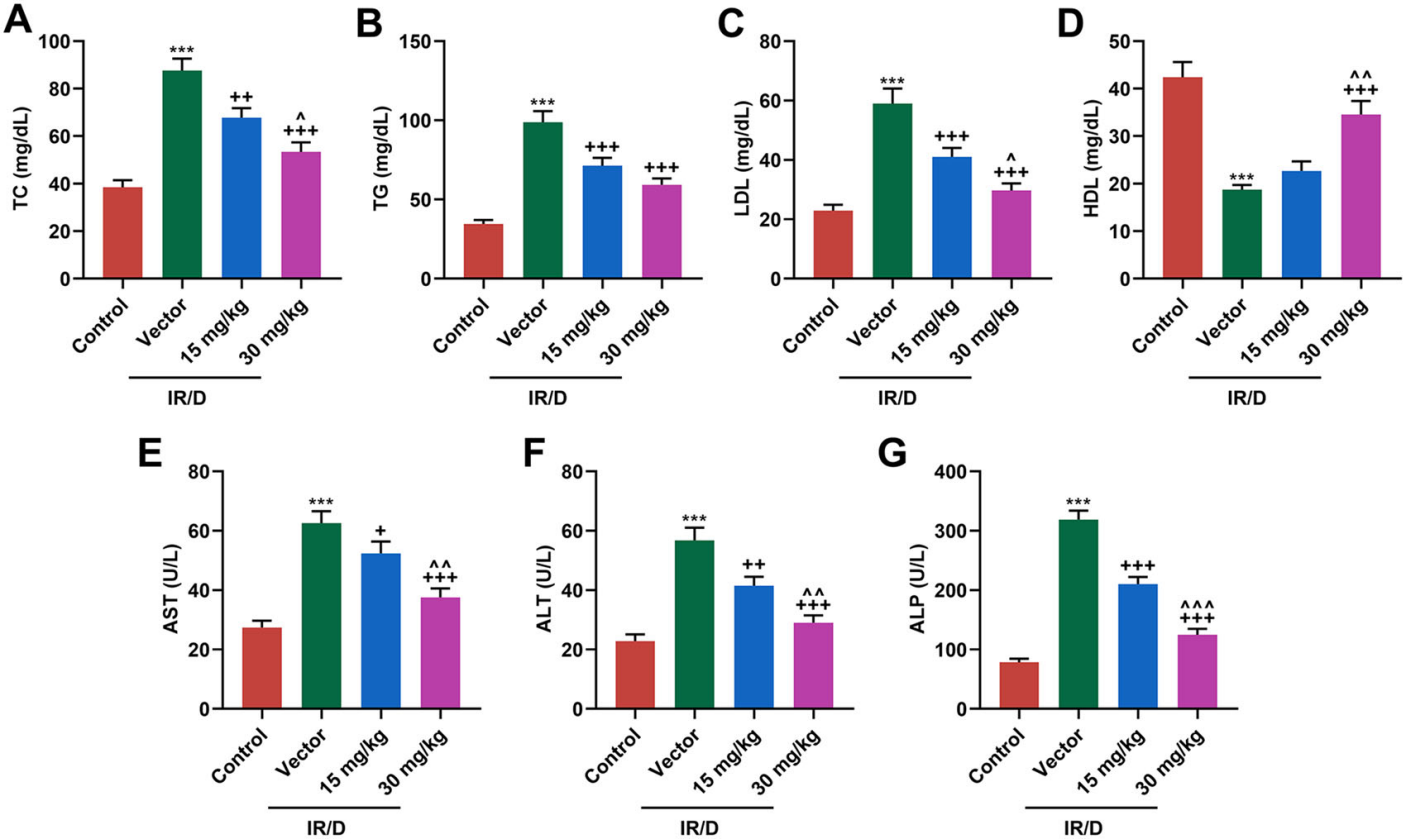
PCA mitigated the abnormal serum levels of liver enzymes and lipid profile in IR/D rats. (A–D) The serum lipid indexes including TC (A), TG (B), LDL (C), and HDL (D) in IR/D rats pretreated with PCA or not were examined by colorimetry assay. (E–G) In IR/D rats pretreated with PCA or not, the serum levels of liver enzymes, including AST (E), ALT (F), and ALP (G), were determined using ELISA. (***p < 0.001 vs. Control; ^++^*p* < 0.01, ^+++^*p* < 0.001 vs. Vector; ^∧^*p* < 0.05, ^∧∧^*p* < 0.01 or ^∧∧∧^*p* < 0.001 vs. 15 mg/kg). (ELISA: Enzyme-linked Immunosorbent Assay, PCA: Protocatechuic Acid, IR/D: insulin resistance type 2 diabetic, TC: total cholesterol, TG: triglycerides, LDL: low density lipids, HDL: high density lipids, AST: aspartate aminotransferase, ALT: alanine aminotransferase, ALP: alkaline phosphatase).

Then, the serum lipid profile in all rats was evaluated. As depicted in Figure 2(A–D), the levels of TC (Figure 2(A)), TG (Figure 2(B)), and LDL (Figure 2(C)) were upregulated, but HDL level (Figure 2(D)) was downregulated in IR/D rats, as compared with those in the control rats (*p* < 0.001). Besides, PCA pretreatment negated these trends in IR/D rats (*p* < 0.01). What’s more, the serum levels of liver enzymes, including AST (Figure 2(E)), ALT (Figure 2(F)), and ALP (Figure 2(G)), were determined. It turned out that IR/D rats had higher levels of serum AST, ALT, and ALP than control rats (*p* < 0.001). Moreover, PCA pretreatment suppressed the upregulation of AST, ALT, and ALP (*p* < 0.001). With the exception of the TG level, 30 mg/kg PCA treatment exerts a stronger effect on the above indicators of IR/D rats than 15 mg/kg PCA treatment (Figure 2, *p* < 0.05). All these discoveries demonstrated that PCA relieved the abnormal serum levels of liver enzymes and lipid profile of IR/D rats.

### PCA ameliorated the oxidative stress, inflammatory response, liver damage, and the downregulated GLP-1 in IR/D rats

**Figure 3.**
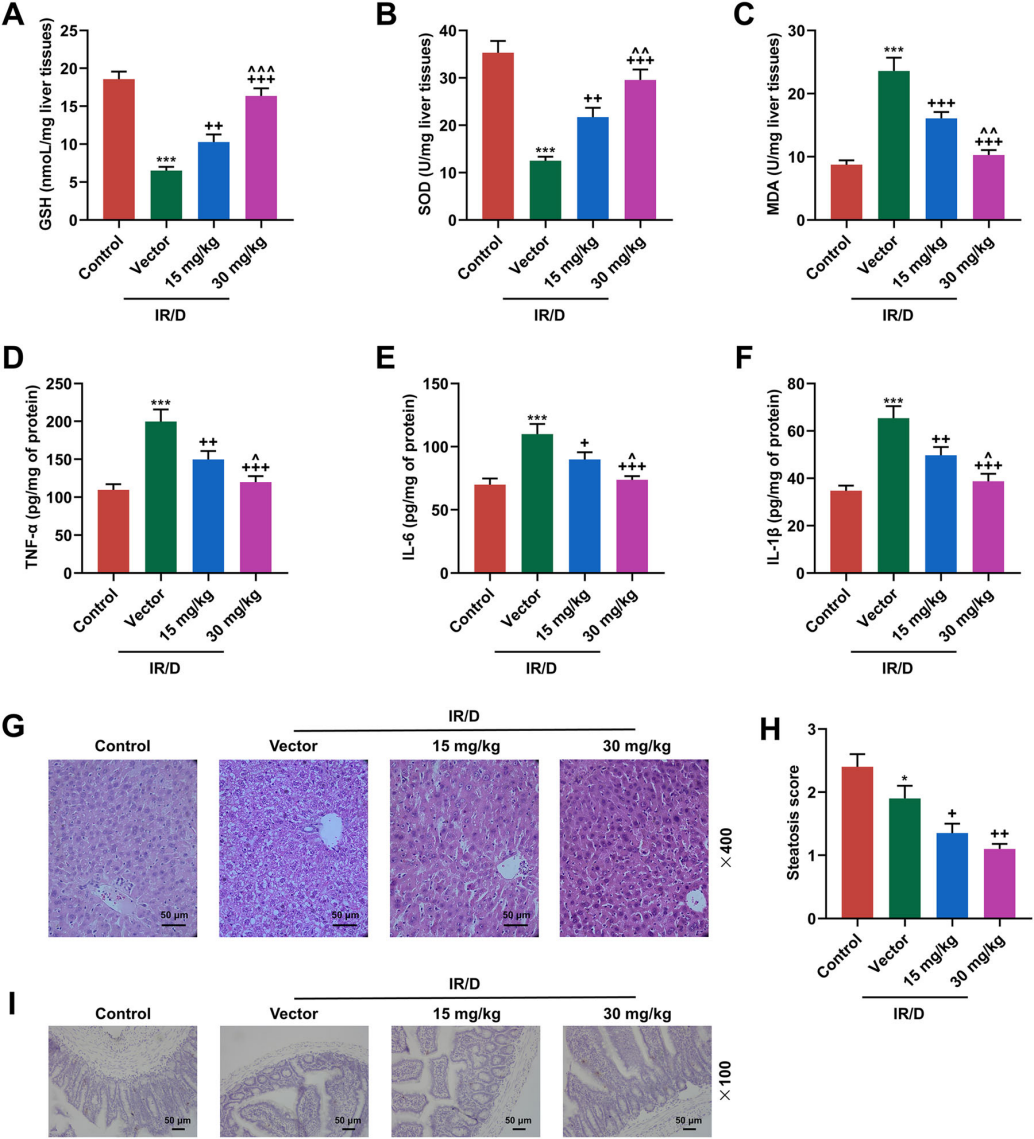
PCA ameliorated the oxidative stress, liver damage, and the downregulated GLP-1 in IR/D rats. (A–C) The antioxidant enzymes and oxidative stress markers including GSH (A), SOD (B), and MDA (C) in the liver tissues of IR/D rats pretreated with PCA or not were examined by ELISA. (D–F) TNF-α, IL-1β and IL-6 in the sera of IR/D rats pretreated with PCA or not were examined by ELISA. (G) The histopathological changes in liver tissues of IR/D rats pretreated with PCA or not were also examined using hematoxylin-eosin staining. (H) Steatosis scores of the liver in different groups. (I) The GLP-1 positive cells in terminal ileum of IR/D rats pretreated with PCA or not were detected by immunohistochemistry. (***p < 0.001 vs. Control; ^++^*p* < 0.01, ^+++^*p* < 0.001 vs. Vector; ^∧^*p* < 0.05, ^∧∧^*p* < 0.01 or ^∧∧∧^*p* < 0.001 vs. 15 mg/kg). (ELISA: Enzyme-linked Immunosorbent Assay, PCA: Protocatechuic Acid, IR/D: insulin resistance type 2 diabetic, GSH: glutathione, SOD: superoxide dismutase, MDA: malondialdehyde, GLP-1: glucagon‐like peptide-1, TNF-α: tumor necrosis factor α).

To evaluate the oxidative stress in liver tissues of IR/D rats, the antioxidant enzymes and oxidative stress markers in liver tissue were evaluated. As exhibited in Figure 3(A–C), IR/D rats exhibited lower levels of GSH and SOD, as well as a higher level of MDA than control rats (*p* < 0.001). Besides, PCA pretreatment exerted opposite impacts on the above aspects in IR/D rat liver tissues (*p* < 0.01). Compared to 15 mg/kg PCA treatment, 30 mg/kg PCA treatment exerts a stronger effect on the levels of GSH, SOD and MDA of IR/D rats (Figure 3(A–C), *p* < 0.01). IR/D rats also exhibited higher levels of pro-inflammatory markers (TNF-α, IL-6 and IL-1β) than control rats (*p* < 0.001), which was revised by PCA treatment at 30 and 50 mg/kg (Figure 3(D–F), *p* < 0.05). Furthermore, the histopathological changes in liver tissues were also examined. As depicted in Figure 3(G), the liver tissue morphology of control rats was observed to be normal, with clear texture and normal central veins. In the vector group, there were hepatocyte hypertrophy injury around the central vein, inflammatory cell infiltration and increased intercellular vacuoles in the rat liver tissues. Post PCA pretreatment, the hepatocyte injury, inflammatory cell infiltration, and increased intercellular vacuoles were ameliorated. Also, the results showed that hepatic steatosis was increased in IR/D rats, whereas treatment with PCA ameliorated hepatic steatosis (Figure 3(H), *p* < 0.05). Additionally, it was further discovered that the GLP-1 positive cells in the terminal ileum of IR/D rats were decreased, whereas PCA pretreatment increased the GLP-1 positive cells in the terminal ileum of IR/D rats (Figure 3(I)). These results indicated that PCA mitigated the oxidative stress, damage, and downregulated GLP-1 in liver tissues of IR/D rats.

### PCA regulated the NF-κB and Wnt1/β-catenin pathways in liver tissues of IR/D rats

**Figure 4.**
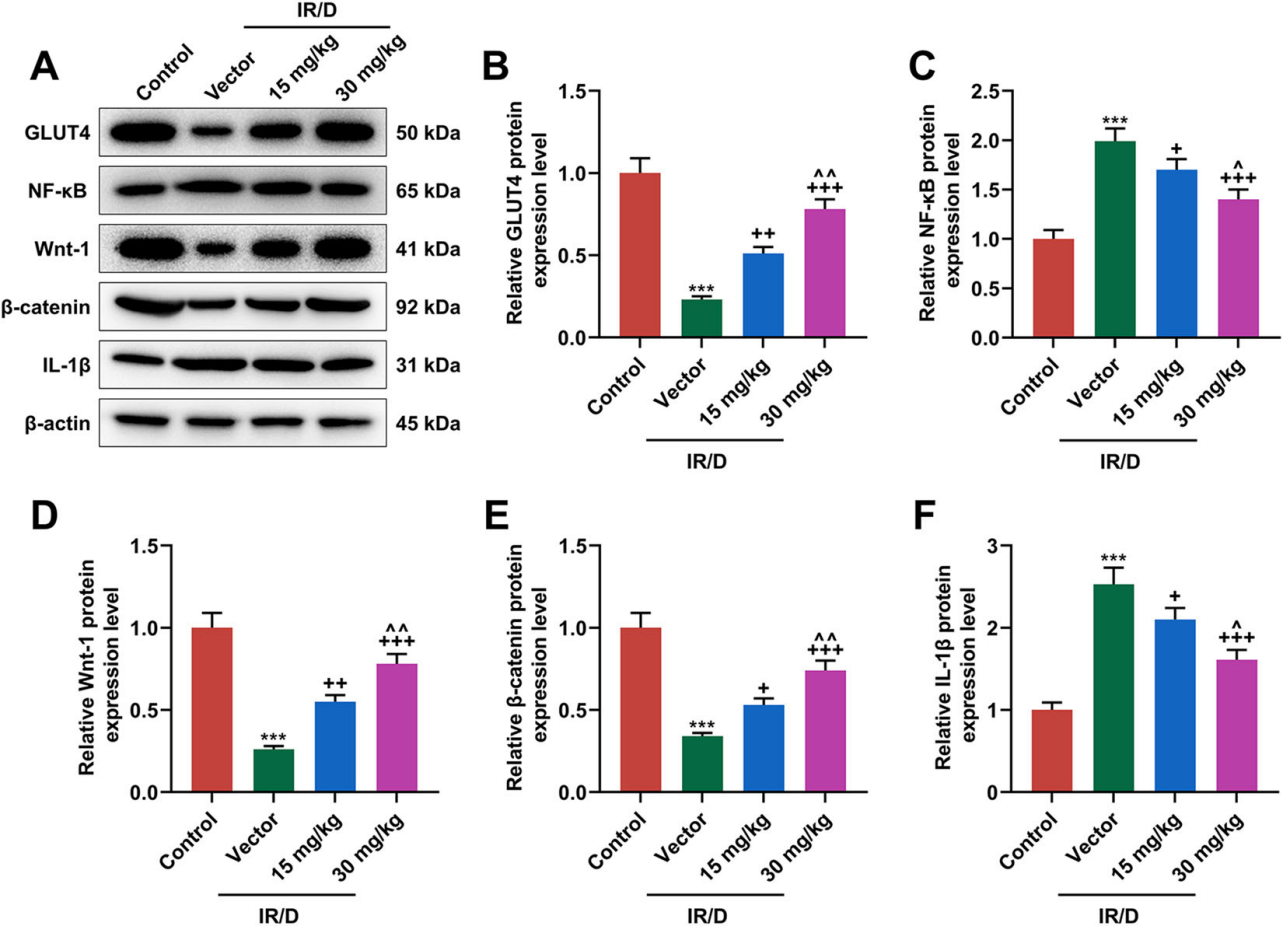
PCA regulated the protein expression of factors related to the NF-κB and Wnt1/β-catenin pathways in liver tissues of IR/D rats. (A–F) The protein expression of GLUT4, NF-κB, Wnt1, β-catenin, and IL-1β in the liver tissues of IR/D rats pretreated with PCA or not was measured by Western blot. (***p < 0.001 vs. Control; ^+^*p* < 0.05, ^++^*p* < 0.01, ^+++^*p* < 0.001 vs. Vector; ^∧^*p* < 0.05 or ^∧∧^*p* < 0.01 vs. 15 mg/kg). (PCA: Protocatechuic Acid, IR/D: insulin resistance type 2 diabetic, IL-1β: interleukin-1β, GLUT4: glucose transporter-4, NF-κB: nuclear factor kappa B).

**Figure 5.**
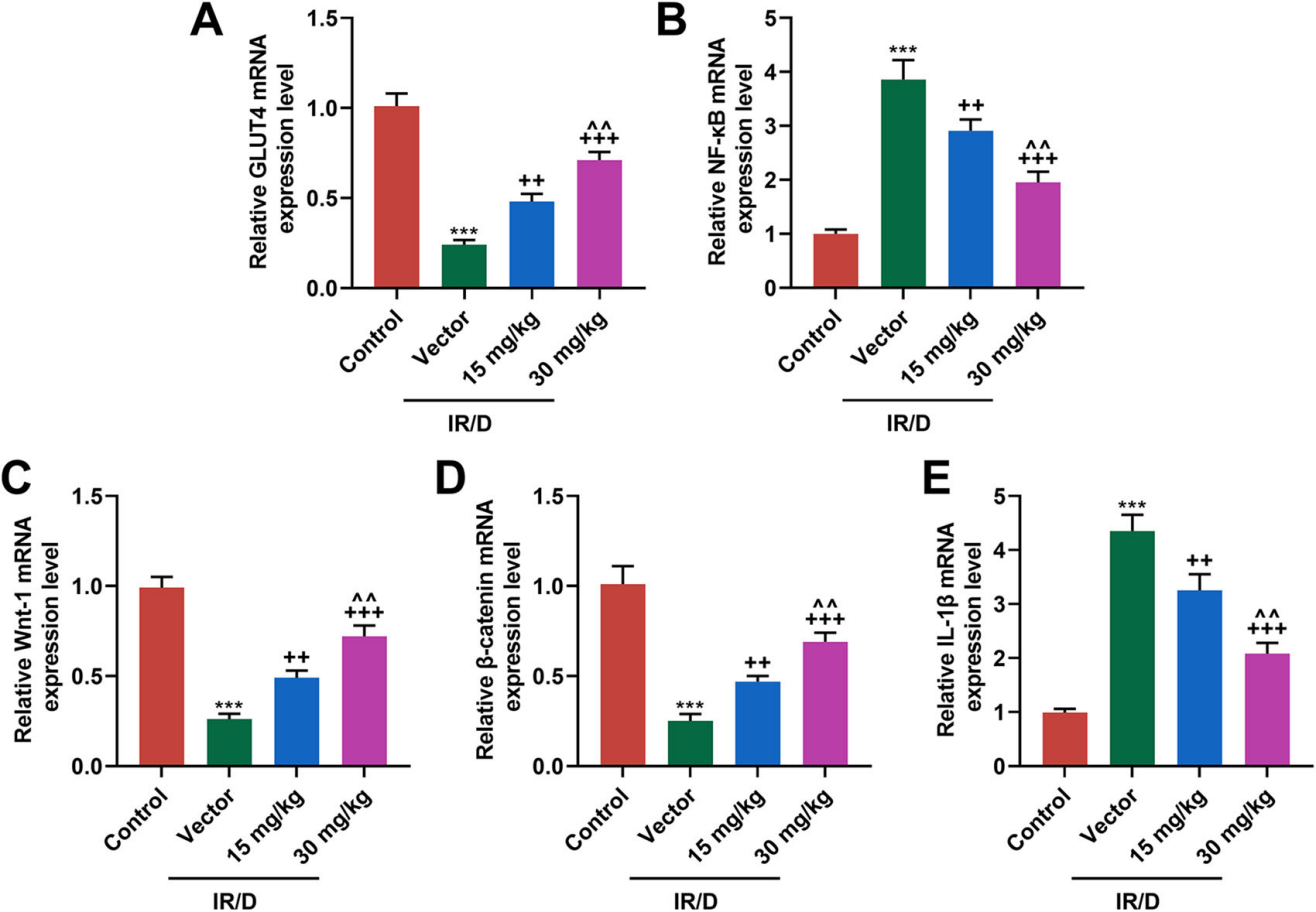
PCA regulated the mRNA expression of factors related to the NF-κB and Wnt1/β-catenin pathways in liver tissues of IR/D rats. (A–E) The mRNA expression of GLUT4, NF-κB, Wnt1, β-catenin, and IL-1β in the liver tissues of IR/D rats pretreated with PCA or not was quantified by qRT-PCR. (***p < 0.001 vs. Control; ^++^*p* < 0.01, ^+++^*p* < 0.001 vs. Vector; ^∧∧^*p* < 0.01 vs. 15 mg/kg). (PCA: Protocatechuic Acid, IR/D: insulin resistance type 2 diabetic, qRT-PCR: quantitative real-time PCR, IL-1β: interleukin-1β, GLUT4: glucose transporter-4, NF-κB: nuclear factor kappa B).

Finally, the potential signal pathway involved in the role of PCA in IR/D rats was determined (Figures 4 and 5). As depicted in Figure 4(A,B,D,E) as well as Figures 5(A,C,D), the protein and mRNA levels of GLUT4, Wnt1, and β-catenin were downregulated in IR/D rats when compared with those in the control rats (*p* < 0.001). PCA pretreatment, on the contrary, increased the GLUT4, Wnt1, and β-catenin levels in IR/D rats (*p* < 0.05). Besides, the expression of NF-κB (Figures 4(C) and 5(B)) and IL-1β (Figures 4(F) and 5(E)) in IR/D rats was upregulated (*p* < 0.001), which however was neutralized by PCA as well (*p* < 0.05). Compared to 15 mg/kg PCA treatment, 30 mg/kg PCA treatment exerts a stronger effect on the above indicators of IR/D rats (Figures 4 and 5, *p* < 0.05). These phenomena signified that PCA regulated the NF-κB and Wnt1/β-catenin pathways in the liver tissues of IR/D rats.

## Discussion

Current management of diabetes is based on synthetic compounds that are commonly associated with severe adverse effects. Compared to synthetic compounds, natural products are relatively non-toxic, inexpensive and available in an ingestible form (Younus and Anwar 2016). Natural products, like polyphenolic compounds derived from plants, modulate blood glucose metabolism through the amplification of cell IR and activation of insulin-like growth factor binding protein signaling pathway (Anwar et al. 2021). PCA is a natural phenolic compound widely existing in medicinal materials, vegetables and fruits, and our research discovered that PCA ameliorated the IR, oxidative stress, lipid accumulation and inflammatory cell infiltration in liver tissues of IR/D rats, and the role of PCA in IR/D rats might be mediated by regulating the NF-κB and Wnt1/β-catenin pathways, signifying PCA as a novel drug candidate for IR/D-induced liver injury.

To investigate the role of PCA in type 2 diabetes-induced liver injury, herein, we constructed the IR/D rat model using HFD long-term administration and streptozotocin single injection, based on the prior researches reporting that chronic administration of HFD causes experimental IR and streptozotocin injection could lead to the destruction of partial pancreatic β cells (Huang et al. 2004; Eleazu et al. 2013). After HFD administration and streptozotocin single injection, the model rats exhibited IR, as evidenced by the higher serum glucose, serum insulin, and HOMA-IR rate, as well as the lower BW level and higher LW and BW/LW levels. Besides, PCA pretreatment upregulated the BW level, downregulated the LW and BW/LW levels, as well as ameliorated the IR of IR/D rats, indicating PCA might have an ameliorating effect on IR/D rats. These above discoveries were analogous to the findings of a previous research that PCA improves the dexamethasone-induced impairment of insulin sensitivity by regulating the serum insulin, glucose, and HOMA-IR value (El-Sonbaty et al. 2019).

Recently, PCA has been reported to yield a hepato-protection effect. For instance, the protective effect of PCA is demonstrated on chemically induced liver fibrosis both *in vitro* and *in vivo* (Cui et al. 2021), and through inhibiting antioxidant, PCA plays a protective effect on the liver of rats with alcoholic liver injury and dexamethasone-induced IR (El-Sonbaty et al. 2019; Fu et al. 2019). In addition, injury and necrosis of hepatocytes are important complications of type 2 diabetes (Bashar et al. 2021). In view of these, we surmised that PCA also had a hepato-protection impact upon IR/D-induced liver injury. It has been elucidated that chronic hyperglycemia in type 2 diabetes has a close relationship with the accumulation of TG and the activation of lipogenic enzymes (Loria et al. 2013; Mota et al. 2016). The serum levels of ALT and AST are the most sensitive indicators of liver damage (Alsahli et al. 2021). Additionally, abnormal liver enzymes and lipid profile are both discovered in IR/D-induced liver injury (Bashar et al. 2021). In the condition of type 2 diabetes, impairment in glucose and lipid metabolism cause the increase of glycogen storage in hepatocytes, which disrupt the efficiency of the mitochondrial electron transport chain and exacerbate oxidative stress in the liver (Yu et al. 2021). Hence, inhibition of glucose, lipid metabolism and oxidative stress is considered to be key in the pathogenesis of diabetic liver injury. In this research, we viewed that IR/D triggered abnormal lipid profile (high levels of TC, TG and LDL; lower level of HDL) and abnormal liver enzymes (upregulation of AST, ALT, and ALP), whilst PCA pretreatment ameliorated the abnormal lipid profile and abnormal liver enzymes, proving that PCA indeed had the hepato-protection effect against IR/D-induced liver injury.

Chronic hyperglycemia promotes the formation of advanced glycation end-products, which are the main source of ROS (Abo El-Nasr et al. 2020). The development of diabetic complications is mediated by the increased oxidative stress and inflammation which are regulated by the interaction of advanced glycation end-products with their receptors (Bierhaus et al. 2005; Lin et al. 2012). In addition, it is widely proven that inflammation and oxidative stress play crucial roles in the development of IR/D-induced liver injury (Mohamed et al. 2016). Notably, previous research pinpointed that PCA can suppress oxidative insults and inflammation in liver and kidney tissues of rats with monosodium glutamate intoxication (Kassab et al. 2022), and also protect the liver against alcoholic liver injury through repressing oxidant pathway (Fu et al. 2019). In this study, we further uncovered that PCA impeded the oxidative stress and inflammation in liver tissues of IR/D rats, with manifestations that PCA mitigated the abnormal oxidative stress markers (GSH, SOD, and MDA) and pro-inflammatory markers (TNF-α, IL-6 and IL-1β), and ameliorated the hepatocyte injury and inflammatory cell infiltration through histomorphological detection. In addition, GLP-1 is synthesized and secreted from the enteroendocrine L-cells to stimulate the secretion of glucose-dependent insulin from β-cells in the pancreas, induce the synthesis of glycogen in the liver, and also improve the oxidation of fatty acids and the removal of lipids from hepatocytes (Bashar et al. 2021). In this research, we also found that in the terminal ileum of IR/D rats, PCA increased the GLP-1-positive cells. All these findings demonstrated that PCA mitigated oxidative stress injury and inflammation in liver tissues of IR/D rats. Mechanically, we then focused on the regulatory pathway *via* which PCA exhibits its role in IR/D-induced liver injury rats.

The increased levels of pro-inflammatory markers, such as TNF-α, C-reactive protein (CRP), IL-1β, IL-6 and ICAM-1 (Intercellular Adhesion Molecule 1) are involved in carbon tetrachloride (CCl_4_)-induced liver injury (Almatroodi et al. 2020). Increased generation of pro-inflammatory cytokine secretion of IL-1β and IL-18 in nonalcoholic steatohepatitis (NASH) mice (Du et al. 2019). Similarly, the levels of NF-κB and IL-1β are upregulated in our IR/D rats. NF-κB signaling pathway has been reported to regulate diabetes mellitus-induced placental or retinal oxidative stress and inflammation (Liu et al. 2020; Shi et al. 2020). Furthermore, NF-κB and Wnt1/β-catenin signaling pathways participate in the regulation of type 2-induced diabetic liver injury in male rats (Bashar et al. 2021). In this study, we observed that PCA pretreatment downregulated NF-κB and IL-1β expression while upregulating the Wnt1, β-catenin, and GLUT4 expression, indicating that the role of PCA in attenuating oxidative stress and inflammation in the liver injury of IR/D rats might be realized by modulating the NF-κB and Wnt1/β-catenin pathways.

Oxidative stress and inflammation are the main causes of tissue damage and highly associated with the incidence of human chronic diseases. The recent study used natural products to inhibit oxidative stress and inflammation so as to ameliorate hepatotoxicity (Alsahli et al. 2021). Antioxidant, anti-inflammatory activities can be implicated in the hepatoprotective potential of PCA *via* modulating mTOR, p53 and the IL-6/IL-17/IL-23 immunoinflammatory pathway (Abdelrahman and El-Tanbouly 2022). Also, PCA-mediated miR-219a-5p activation alleviates alcoholic liver injury by inhibiting the p66shc oxidant pathway (Fu et al. 2019). PCA is therefore a potential therapeutic candidate for improving diabetic hepatic injury through exhibiting antioxidant and anti-inflammation activities. Also, the observed effects of PCA treatment would be an excellent way to prevent damage from acute oxidative stress.

Collectively, PCA ameliorates the IR, oxidative stress, and inflammation in liver tissues of IR/D rats, which might be achieved by regulating the NF-κB and Wnt1/β-catenin pathways. These findings hint PCA as a promising novel drug candidate and provide research direction for the prevention and treatment of IR/D-induced liver injury to a certain extent. Available findings are limited to just animal research. Future explorations are necessary to reveal the safety and therapeutic efficacy of PCA in the clinic.

## Data Availability

The analyzed data sets generated during the study are available from the corresponding author on reasonable request.
